# Combined Use of Diffusion- and Perfusion-Weighted Magnetic Resonance Imaging in the Differential Diagnosis of Sellar Tumors: A Single-Centre Experience

**DOI:** 10.3390/jcm14207168

**Published:** 2025-10-11

**Authors:** Adrian Korbecki, Marek Łukasiewicz, Arkadiusz Kacała, Michał Sobański, Agata Zdanowicz-Ratajczyk, Karolina Szałata, Mateusz Dorochowicz, Justyna Korbecka, Grzegorz Trybek, Anna Zimny, Joanna Bladowska

**Affiliations:** 1 Department of General and Interventional Radiology and Neuroradiology, Medical University Hospital, 50-556 Wroclaw, Poland; 2 Hetalox sp. z o.o., 54-105 Wroclaw, Poland; 3 Lower Silesian Specialist Hospital T. Marciniak—Emergency Medicine Center, 54-049 Wroclaw, Poland; 4 Department of Radiology, Wroclaw Medical University, 50-367 Wroclaw, Poland; 5 Department of Neurology, Wroclaw Medical University, 50-367 Wroclaw, Poland; 6 4th Military Clinical Hospital in Wroclaw, Rudolfa Weigla 5, 50-981 Wroclaw, Poland; 7 Department of Oral Surgery, Pomeranian Medical University in Szczecin, 70-111 Szczecin, Poland; 8 Faculty of Medicine, Wroclaw University of Science and Technology, 50-370 Wroclaw, Poland; 9 Department of Radiology, 4th Military Clinical Hospital with Polyclinic, Independent Public Healthcare Institution, 50-981 Wroclaw, Poland

**Keywords:** pituitary neuroendocrine tumors (PitNETs), sellar and parasellar tumors, diffusion-weighted imaging (DWI), perfusion-weighted imaging (PWI), apparent diffusion coefficient (ADC), differential diagnosis, Magnetic Resonance Imaging (MRI)

## Abstract

**Background/Objectives:** To evaluate whether incorporating both diffusion-weighted imaging (DWI) and perfusion-weighted imaging (PWI) in pituitary MRI examinations improves differential diagnosis by providing additional diagnostic value. **Methods:** A retrospective analysis was performed on 88 patients with histologically confirmed sellar or parasellar tumors who underwent 1.5T MRI with DWI and dynamic susceptibility contrast PWI (DSC-PWI) between October 2007 and April 2023. DWI parameters included minimum apparent diffusion coefficient (ADCmin) and relative ADCmin (rADCmin). PWI parameters included mean and maximum relative cerebral blood volume (rCBV, rCBVmax) and relative peak height (rPH, rPHmax), normalized to white matter. Tumor regions of interest were manually segmented, excluding calcified or hemorrhagic areas. Group comparisons and ROC analyses assessed diagnostic performance of individual and combined parameters. **Results:** Significant differences in diffusion and perfusion metrics were observed among the five tumor types. The combined analysis of DWI and PWI improved diagnostic accuracy in selected comparisons. The greatest benefit occurred in distinguishing meningiomas from solid non-functional pituitary adenomas (pituitary neuroendocrine tumors-PitNET), where the combination of ADCmin and rPHmax yielded an AUC of 0.818, sensitivity of 88%, and specificity of 76%, exceeding the performance of either parameter alone. In other comparisons, including meningiomas versus invasive PitNETs and adamantinomatous craniopharyngiomas, combined analysis did not substantially improve accuracy when single parameters, particularly rCBVmax (AUC = 0.995), already demonstrated excellent performance. **Conclusions:** Integration of DWI and PWI into pituitary MRI protocols enhances diagnostic performance in selected tumor groups. The additive value is context-dependent, supporting the tailored application of these sequences in the evaluation of sellar and parasellar tumors.

## 1. Introduction

Tumors of the sellar and parasellar regions represent a heterogeneous group of primary intracranial neoplasms, accounting for approximately 10–15% of all central nervous system (CNS) tumors [1]. The most common entities include adenomas (pituitary neuroendocrine tumors-PitNET), meningiomas, and craniopharyngiomas as well as Rathke’s cleft cysts, while less frequent lesions comprise gliomas, metastases, and abscesses [1,2]. These tumors often present with overlapping clinical and radiological features, making their preoperative differentiation challenging in many cases.

Magnetic resonance imaging (MRI) remains the primary imaging modality for assessing sellar and parasellar lesions. Conventional MRI sequences are often sufficient for identifying larger tumors or those with characteristic features, such as the cystic components of craniopharyngiomas or calcifications seen on CT. However, these standard protocols may not reliably distinguish between lesions with more subtle differences in tissue characteristics or growth patterns [3,4].

Accurate preoperative differentiation of pituitary region tumors is critical, as it directly informs the neurosurgical approach, treatment strategy, and impacts both the chance of recurrence and the required frequency of follow-up [5,6]. For instance, prolactinomas can often be effectively managed with dopamine agonists, and misdiagnosing them as non-functional adenomas may lead to unnecessary surgery [7]. Conversely, invasive adenomas may require more aggressive resection and adjuvant radiotherapy, particularly in cases with cavernous sinus involvement, where transsphenoidal resection is incomplete. Similarly, misclassifying an adamantinomatous craniopharyngioma as a macroadenoma may result in an inappropriate transsphenoidal approach instead of a craniotomy. Furthermore, meningiomas—although they often encase the internal carotid artery—may be misdiagnosed as invasive adenomas in cases where such invasion is not clearly evident [5,6,7].

Differentiation also allows neurosurgeons to anticipate tumor consistency and vascularity, helping to plan for potential intraoperative difficulties such as bleeding or firm lesion dissection. Identifying high-perfusion lesions like meningiomas in advance can improve operative safety. Ultimately, precise diagnosis enables better surgical planning, guides decisions regarding the extent of resection, and facilitates timely decisions regarding the use of adjuvant therapies such as radiotherapy.

Advanced MRI techniques, including diffusion-weighted imaging (DWI) and perfusion-weighted imaging (PWI), offer additional physiologic insights into tumor cellularity and vascularity, respectively. While DWI and PWI are routinely employed in neuro-oncology, their use in pituitary tumor imaging remains limited, inconsistently implemented, and underrepresented in clinical guidelines [8,9,10]. In particular, dynamic susceptibility contrast (DSC) PWI has been scarcely studied in the context of sellar tumors.

The purpose of this study was to evaluate whether the combined use of DWI and PWI can enhance the diagnostic differentiation of common sellar and parasellar tumors—specifically non-functional solid adenomas, invasive adenomas, meningiomas, prolactinomas, and adamantinomatous craniopharyngiomas. By improving preoperative tumor characterization, we aim to support more accurate clinical decision-making and surgical planning.

## 2. Materials and Methods

### 2.1. Study Population

This study was approved by the Institutional Review Board of the local Ethics Committee, which oversees research involving human participants. It was conducted at a single center and included 88 patients diagnosed with sellar or parasellar tumors who underwent MRI examinations incorporating both DWI and DSC PWI. The study period spanned from October 2007 to April 2023.

The study cohort comprised 43 women (48.9%) and 45 men (51.1%), with a mean age of 59 years (SD ± 15 years). All 88 examinations were classified into five tumor categories: non-functional solid adenomas (25 cases, 28.4%), invasive pituitary adenomas (22 cases, 25.0%), meningiomas (16 cases, 18.2%), prolactinomas (13 cases, 14.8%), and adamantinomatous craniopharyngiomas (12 cases, 13.6%). Invasive adenomas were identified based on the criteria described by Hansen et al. [11]

Examinations were excluded if the tumor measured less than 1.0 cm in both the anterior–posterior (AP) and craniocaudal (CC) dimensions (i.e., less than twice the slice thickness), or if significant artifacts from calcifications within the lesion were present. However, in rare cases where such small lesions were fully visualized within the imaging slice and allowed for accurate measurement, they were retained in the analysis. Detailed characteristics of the study group are presented in Table 1.

### 2.2. MRI Acquisition and Processing

All examinations were performed on a 1.5T GE Signa Hdx scanner (GE Healthcare, Milwaukee, WI, USA) using a 16-channel head, neck, and spine coil. The standard pituitary MRI protocol included multiplanar T1- and T2-weighted sequences, as well as axial 3D T1-weighted sequences acquired after intravenous administration of gadolinium-based contrast. Conventional sequences were used to assess lesion dimensions (AP, transverse [TR], and CC diameters), morphology (solid or cystic components), and the presence of hemorrhage or calcifications.

#### 2.2.1. Diffusion-Weighted Imaging

**Figure 1:**
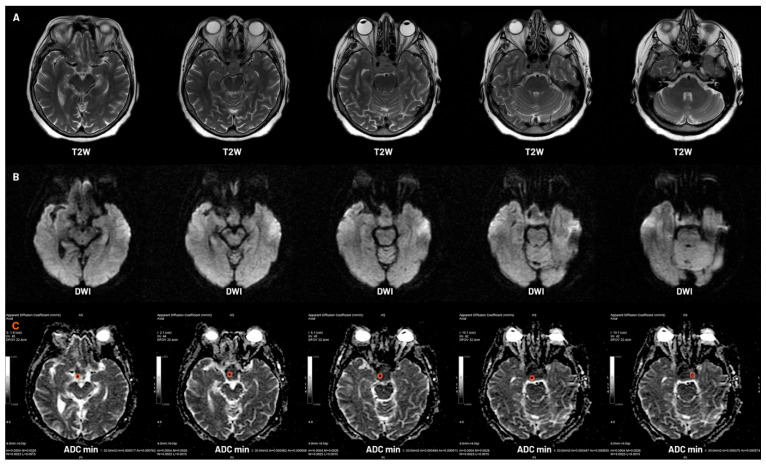
MRI evaluation of a sellar region lesion using conventional and diffusion-weighted imaging. (**A**) Axial T2-weighted images showing an invasive adenoma. (**B**) Corresponding diffusion-weighted imaging (DWI) sequences demonstrating restricted diffusion within the lesion. (**C**) Apparent diffusion coefficient (ADC) maps with ROI-based measurements of ADCmin across consecutive axial slices, indicating areas of lowest diffusivity.

DWI was performed using a single-shot spin-echo echo-planar imaging sequence with two b-values: 0 and 1000 mm^2^/s. Acquisition parameters included: TR = 10,000 ms, TE = 82.7 ms, field of view (FOV) = 24 cm, matrix = 128 × 128, slice thickness = 5 mm, interslice gap = 1 mm, and number of excitations (NEX) = 5. DWI data were processed on a GE Advantage Workstation (AW, version 4.6; GE Medical Systems) with Functool software (GE Healthcare Functool; available online: https://www.gehealthcare.com, accessed on 1 September 2025). Absolute apparent diffusion coefficient (ADC) values were measured as the mean ADC and minimum ADC (ADCmin) within the tumor (Figure 1). Relative ADC values (mean rADC and rADCmin) were calculated by dividing tumor ADC values by those from normal-appearing white matter. For subsequent analysis, only ADCmin and rADCmin values were retained. Measurement methodology followed protocols described in our prior work on pituitary DWI [12].

#### 2.2.2. Dynamic Susceptibility Contrast Perfusion-Weighted Imaging

PWI was performed using a T2*-weighted gradient-echo echo-planar imaging sequence with the following parameters: TR = 1900 ms, TE = 80 ms, FOV = 30 cm, flip angle = 90°, matrix = 192 × 129, slice thickness = 8 mm. Gadolinium-based contrast (0.2 mmol/kg) was injected at 5 mL/s through an antecubital vein, followed by a 20 mL saline flush at the same rate. Acquisition commenced 10 s before contrast injection to establish a baseline. No pre-bolus technique was used.

Perfusion data were processed using ReadyView software (GE Healthcare, GE Medical Systems; available online: https://www.gehealthcare.com, accessed on 1 September 2025). Normalization was performed, and color-coded cerebral blood volume (CBV) maps were generated. Baseline signal intensity (S0), minimum signal intensity during contrast passage (Smin), and post-contrast recovery signal intensity (S1) were measured in normal-appearing white matter to calculate peak height (PH) and percentage signal recovery (PSR). These reference values were used to derive relative tumor metrics.

**Figure 2:**
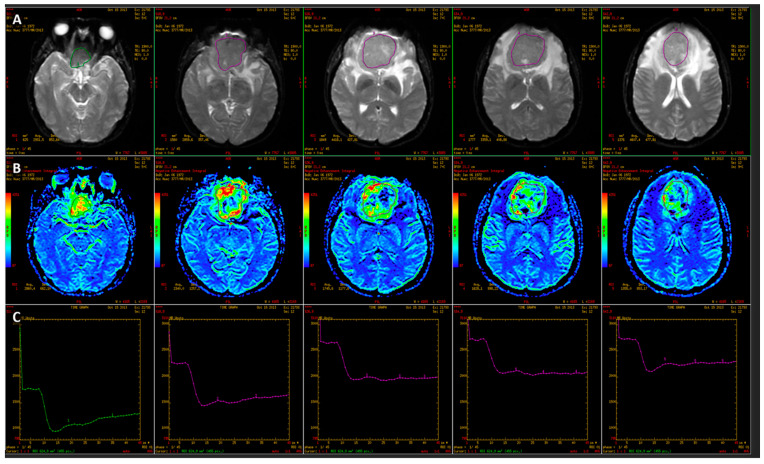
Perfusion-weighted imaging (PWI) analysis of a large parasellar meningioma. (**A**) Axial T2-weighted images showing the extent of the solid lesion with manually defined ROIs. (**B**) Corresponding perfusion maps demonstrating markedly elevated perfusion within the lesion, particularly in its anterior and peripheral regions. (**C**) Signal intensity–time curves derived from dynamic susceptibility contrast (DSC) perfusion sequences, showing a characteristic drop in signal intensity followed by partial recovery, consistent with high vascularity.

Sellar and parasellar lesions were manually segmented on all axial sections, excluding regions with calcification, hemorrhage, or adjacent vascular structures (Figure 2). Relative perfusion parameters—rCBV, rPH, and maximum rCBV (rCBVmax), maximum rPH (rPHmax)—were calculated by dividing tumor values by those from normal-appearing white matter. Metrics were recorded both as the mean value across all tumor slices (mean rCBV) and as the highest value from any slice (rCBVmax). The rPSR parameter was excluded from further statistical analysis due to its lack of significant contribution [10].

### 2.3. Statistical Analysis

All statistical analyses were performed using R software (version 4.1.2; R Foundation for Statistical Computing, Vienna, Austria). Categorical variables were summarized as counts and percentages, while continuous variables were described using descriptive statistics. The normality of continuous variable distributions was assessed using the Shapiro–Wilk test and by evaluating skewness and kurtosis. Normally distributed variables were reported as mean ± SD, and non-normally distributed variables were reported as median with interquartile range (IQR; first and third quartiles).

Comparisons of continuous variables among tumor types were conducted using one-way analysis of variance (ANOVA) for normally distributed data or the Kruskal–Wallis test for non-normally distributed data. When overall group differences were statistically significant, post hoc pairwise comparisons were performed using Tukey’s honestly significant difference test for ANOVA or Dunn’s test with Bonferroni correction for the Kruskal–Wallis test.

The diagnostic performance of MRI-derived parameters—evaluated individually and in combination—was assessed using receiver operating characteristic (ROC) curve analysis for tumor type pairs. Only tumor categories with more than 10 cases were included in ROC analysis. The optimal cutoff values for each parameter were determined using Youden’s index. For each parameter and parameter combination, the area under the ROC curve (AUC), sensitivity, specificity and accuracy were calculated. For parameter combinations, a binary variable was created based on the optimal cutoff values for each DWI and PWI metric (as determined by Youden’s index), and ROC analysis was performed on this variable to assess combined diagnostic performance, while preserving interpretability of individual thresholds.

All statistical tests were two-tailed, and a *p*-value < 0.05 was considered to indicate statistical significance.

## 3. Results

The descriptive statistics for diffusion- and perfusion-derived parameters across the five tumor groups are summarized in Table 2.

For ADCmin values, adamantinomatous craniopharyngiomas demonstrated the highest mean value (1.45 ± 0.36 × 10^−3^ mm^2^/s; median = 1.42, IQR = 1.29–1.70), while invasive pituitary adenomas showed the lowest (0.58 ± 0.10; median = 0.56, IQR = 0.52–0.64).

Similarly, for rADCmin, craniopharyngiomas had the highest values (1.83 ± 0.44; median = 1.92, IQR = 1.56–2.23) and invasive adenomas the lowest (0.74 ± 0.12; median = 0.73, IQR = 0.67–0.81).

Among perfusion parameters, rCBVmax was greatest in meningiomas (7.05 ± 3.34; median = 5.82, IQR = 5.17–7.41) and lowest in adamantinomatous craniopharyngiomas (2.22 ± 0.95; median = 2.15, IQR = 1.84–2.54). A similar pattern was observed for rCBV, with meningiomas showing the highest values (5.23 ± 2.16; median = 4.53, IQR = 3.90–6.32) and craniopharyngiomas the lowest (1.52 ± 0.77; median = 1.39, IQR = 1.22–1.84).

For rPHmax, meningiomas again demonstrated the highest mean value (3.43 ± 1.99; median = 3.00, IQR = 2.28–4.50), while craniopharyngiomas had the lowest (1.80 ± 0.89; median = 1.80, IQR = 1.45–2.05).

Similarly, mean rPH values were greatest in meningiomas (2.26 ± 1.28; median = 2.10, IQR = 1.49–2.45) and lowest in craniopharyngiomas (0.89 ± 0.50; median = 0.77, IQR = 0.47–1.24).

For tumors pairs in which both DWI and PWI parameters demonstrated statistically significant differences, ROC curve analysis was performed to assess the combined diagnostic performance of these metrics. Three tumor pairs met the required criteria: meningiomas vs. non-functional solid adenomas, meningiomas vs. invasive pituitary adenomas, and meningiomas vs. adamantinomatous craniopharyngiomas.

For differentiation between meningiomas and non-functional solid adenomas, the most effective parameter combination was ADCmin and rPHmax, achieving an AUC of 0.818 (95% CI: 0.690–0.920), with a sensitivity of 88% and specificity of 76%. In comparison, ADCmin alone showed perfect sensitivity (100%) but low specificity (32%), while rPHmax alone yielded an AUC of 0.740 (95% CI: 0.578–0.880), with a sensitivity of 88% and specificity of 60%. The combined parameters improved the overall diagnostic balance, reaching an accuracy of 80%.

For differentiation between meningiomas and invasive pituitary adenomas, the combination of ADCmin and rCBV provided the best trade-off between sensitivity and specificity, with an AUC of 0.830 (95% CI: 0.699–0.946), sensitivity of 75%, and specificity of 91%. ADCmin alone demonstrated the highest AUC of 0.872 (95% CI: 0.743–0.972), with balanced sensitivity (81%) and specificity (82%). The pairing of ADCmin and rCBVmax achieved an AUC of 0.815 (95% CI: 0.676–0.923), maintaining the same sensitivity and specificity profile as the ADCmin alone. Relative diffusion parameters, including rADCmin combined with rCBV (AUC = 0.767; 95% CI: 0.633–0.892) and with rCBVmax (AUC = 0.753; 95% CI: 0.608–0.892), provided similar performance, with slightly lower sensitivity but specificity reaching up to 91%.

For differentiation between meningiomas and adamantinomatous craniopharyngiomas, the highest diagnostic accuracy was obtained using the combination of rADCmin and rCBV, yielding an AUC of 0.906 (95% CI: 0.812–1.000), with sensitivity of 81% and specificity of 100%. Comparable performance was seen for rADCmin combined with rCBVmax (AUC = 0.906; 95% CI: 0.812–1.000). rADCmin and rPH also demonstrated excellent diagnostic value, achieving an AUC of 0.844 (95% CI: 0.719–0.938), sensitivity of 69%, and specificity of 100%. Notably, individual rCBV and rCBVmax metrics alone reached near-perfect diagnostic performance, with AUCs of 0.984 and 0.995, respectively.

Precise cut-off values, along with corresponding AUC, sensitivity, specificity, accuracy, and *p*-values for all individual and combined DWI and PWI parameters, are presented in Table 3. These thresholds may serve as a reference for diagnostic decision-making.

## 4. Discussion

To date, numerous studies have demonstrated the diagnostic value of DWI or PWI individually in the evaluation of sellar and parasellar tumors [9,10,13,14]. DWI-based ADC analysis has shown potential in distinguishing pituitary adenomas from other lesion types, while PWI-derived CBV parameters have been used, e.g., to differentiate sellar meningioms [10,14,15,16,17]. However, to our knowledge, no previous study has systematically evaluated the combined use of DWI and PWI in a comprehensive, integrated diagnostic framework for tumors of the pituitary region.

In contrast, the combined application of DWI and PWI has been well established in the broader context of differential diagnosis of intracranial neoplasms, prognostic and therapeutic relevance in radiotherapy planning and stratifying patients based on treatment response [18,19,20,21,22]. These advanced imaging techniques provide complementary information: DWI reflects tumor cellularity, whereas PWI characterizes vascularity and hemodynamics [9,23]. Such multiparametric approaches have been shown to improve diagnostic accuracy beyond conventional T1- and T2-weighted imaging, which often fails to adequately discriminate among overlapping entities [18].

Motivated by this evident gap in the literature on pituitary imaging, our study aimed to assess whether a combined analysis of diffusion and perfusion parameters can enhance the differential diagnosis of sellar and parasellar tumors.

Based on our previous research [10,12,17], the current study involved a targeted statistical analysis to identify which tumors exhibited significant differences in both DWI and PWI parameters. The following tumor pairs were selected for further analysis to evaluate whether a combined approach—integrating both DWI- and PWI-derived metrics—could enhance diagnostic discrimination: meningiomas versus solid non-functional adenomas, meningiomas versus invasive pituitary adenomas, and meningiomas versus adamantinomatous craniopharyngiomas. This combined analysis was conducted solely to determine whether simultaneous use of cut-off values from both sequences in specific differential diagnoses offers added diagnostic value over using them individually.

Distinguishing meningiomas from non-functional solid adenomas, the most effective combination was ADCmin and rPHmax, which yielded an AUC of 0.818 (95% CI: 0.690–0.920), with a sensitivity of 88% and specificity of 76%. This dual-parameter analysis outperformed the isolated use of ADCmin (AUC = 0.675, specificity = 32%) and rPHmax (AUC = 0.740, specificity = 60%). The isolated DWI and PWI findings align with previous literature, where solid adenomas are known to exhibit lower ADC values due to higher cellularity, while meningiomas, as extra-axial tumors, show significantly higher perfusion compared to adenomas, which may also demonstrate elevated perfusion. [10,24,25]. The joint analysis combining ADCmin (cut-off: 0.54) and rPHmax (cut-off: 1.62) proved beneficial, offering a more balanced and clinically useful diagnostic performance.

In contrast, for other tumors pairs, including meningiomas vs. invasive pituitary adenomas and meningiomas vs. adamantinomatous craniopharyngiomas, we observed that while combined analyses showed high diagnostic value, they did not significantly outperform the best single-parameter models. For instance, in the differentiation of meningiomas vs. invasive adenomas, the combination of ADCmin and rCBV reached an AUC of 0.830 (95% CI: 0.699–0.946). However, ADCmin alone already achieved an AUC of 0.872 (95% CI: 0.743–0.972), with comparable sensitivity (81%) and specificity (82%). Similarly, in the comparison between meningiomas and adamantinomatous craniopharyngiomas, the combination of rADCmin and rCBV yielded an AUC of 0.906, while rADCmin alone reached 0.964, and rCBV alone even 0.984—suggesting that isolated use of DWI and PWI parameters alone may sufficiently discriminate in this context. The specific cut-off values for ADC and PWI parameters used in these comparisons are provided in Table 3.

In the case of meningiomas and invasive adenomas, both tumor types may present with overlapping features on conventional MRI, often making differentiation challenging based on morphology alone [2,26]. However, invasive adenomas are known to exhibit significantly lower ADCmin and rADCmin values due to higher cellularity [24,27,28]. This makes DWI parameters particularly valuable in distinguishing these two entities. In contrast, perfusion imaging does not offer comparable discriminatory power in this context, and its addition to DWI-based analysis does not result in improvement in diagnostic performance [10,17].

For meningiomas versus adamantinomatous craniopharyngiomas, the situation is different. These tumors typically exhibit distinct morphological features on conventional MRI: adamantinomatous craniopharyngiomas are often mixed cystic and solid lesions, while meningiomas present as uniformly solid, extra-axial masses [2,26]. As a result, both DWI and PWI parameters alone are sufficient to achieve high diagnostic accuracy [10,29,30]. For example, perfusion metrics such as rCBVmax already yield near-perfect classification in this comparison. In such cases, where single-modality metrics already demonstrate excellent performance, the incremental value of combined DWI–PWI analysis is limited, particularly when conventional imaging sequences already provide strong diagnostic clues. Additionally, it is worth noting that when individual parameters already demonstrate high AUC, specificity, and sensitivity, combining them may not provide added diagnostic value. In such cases, the combined model does not outperform the isolated metrics, as has also been observed in other intracranial pathologies [31].

Notably, we also observed a general trend in diagnostic performance across modalities. DWI-derived metrics, particularly ADCmin, tended to provide more balanced sensitivity and specificity, making them reliable in broader diagnostic scenarios. A similar trend has been observed in other studies, both for pituitary lesions and other intracranial neoplasms [12,32]. In contrast, PWI-derived parameters often showed higher sensitivity but lower specificity, which may lead to false positives if used in isolation. Thus, the clinical application of these metrics may depend on whether the diagnostic goal is to rule in or rule out a given tumor type, with DWI potentially favoured in uncertain cases requiring specificity, and PWI aiding in initial sensitive detection.

This study has several limitations. First, all examinations were performed using a 1.5T MRI scanner, which may have reduced the spatial resolution and signal-to-noise ratio compared to 3T systems. However, the use of a single scanner across all patients ensured consistency in image acquisition and allowed for reliable longitudinal data collection over several years. Additionally, we utilized a standard DWI protocol typically included in routine head MRI exams, enhancing the clinical applicability of our approach.

Second, we excluded small lesions and tumors with calcifications or hemorrhagic components, in line with previous studies [10,12]. In these cases, accurate and artifact-free measurements of perfusion and diffusion parameters were not feasible due to the limitations of both DWI and PWI in regions with susceptibility artifacts. Consequently, our findings apply primarily to larger, non-calcified, and non-hemorrhagic lesions.

Third, while our study included 88 patients, a larger and more histologically diverse cohort would have increased statistical power and may have uncovered additional diagnostic differences between tumor types. Future studies with expanded populations and stratified subgroups could help validate and extend our findings.

In conclusion, our results support the clinical value of performing both DWI and PWI sequences in the evaluation of pituitary region tumors. However, these sequences should be analyzed individually, guided by knowledge of which modality is most informative for specific tumor types. Such a tailored approach can enhance diagnostic precision and help to extract the most relevant information from each imaging modality. Importantly, DWI adds only about one minute to the scan time, and gadolinium contrast is administered routinely regardless of PWI, meaning no additional contrast burden is placed on the patient.

## Figures and Tables

**Table 1:** The quantitative features of the study.

Patient Age	n	M ± SD	Me (Q1; Q3)	Range
Age	88	59.28 ± 15.79	63.50 (51.00; 68.25)	21.00–86.00
Tumor size	n	M ± SD	Me (Q1; Q3)	Range
TR	88	2.79 ± 1.11	2.70 (2.00; 3.50)	0.70–6.60
AP	88	2.58 ± 1.02	2.40 (1.80; 3.30)	1.20–5.90
CC	88	2.82 ± 1.33	2.65 (1.78; 3.52)	0.60–7.30
volume	88	14.64 ± 16.13	9.61 (3.44; 20.70)	0.35–77.12
Tumor type	n	%		
Non-functional solid adenomas	25	28.4		
Invasive pituitary adenomas	22	25		
Meningiomas	16	18.2		
Prolactinomas	13	14.8		
Adamantinomatous craniopharyngiomas	12	13.6		
DWI and PWI parameters:	n	M ± SD	Me (Q1; Q3)	Range
ADCmin	88	0.76 ± 0.35	0.66 (0.54; 0.83)	0.37–1.96
rADCmin	88	0.96 ± 0.42	0.84 (0.69; 1.08)	0.49–2.38
rCBV	88	3.08 ± 1.93	2.69 (1.48; 4.32)	0.29–11.33
rPH	88	1.75 ± 1.24	1.48 (0.76; 2.26)	0.20–5.92
rCBVmax	88	4.15 ± 2.63	3.78 (2.17; 5.40)	0.42–17.77
rPHmax	88	2.55 ± 1.65	2.08 (1.37; 3.35)	0.29–8.16

Abbreviations: ADC—apparent diffusion coefficient; rADC—relative apparent diffusion coefficient; rCBV—relative cerebral blood volume; rPH—relative peak height; TR—transverse dimension; AP—anteroposterior dimension; CC—craniocaudal dimension; min—minimum; max—maximum; M—mean; SD—standard deviation; Me—median; Q1—first quartile; Q3—third quartile.

**Table 2:** Statistics for ADCmin, rADCmin, rCBV, rCBVmax, rPH, and rPHmax values across pituitary and parasellar tumor types. Values are presented as mean ± standard deviation (M ± SD), median (first quartile; third quartile), and range.

Variable	Group	n	M ± SD	Me (Q1; Q3)	Range
ADCmin	Non-functional solid adenomas	25	0.67 ± 0.18	0.70 (0.53; 0.76)	0.37–1.06
ADCmin	Invasive pituitary adenomas	22	0.58 ± 0.10	0.56 (0.52; 0.64)	0.43–0.92
ADCmin	Meningiomas	16	0.78 ± 0.19	0.73 (0.65; 0.84)	0.55–1.20
ADCmin	Prolactinomas	13	0.60 ± 0.20	0.57 (0.44; 0.67)	0.39–1.00
ADCmin	Adamantinomatous craniopharyngiomas	12	1.45 ± 0.36	1.42 (1.29; 1.70)	0.87–1.96
rADCmin	Non-functional solid adenomas	25	0.85 ± 0.22	0.87 (0.69; 0.97)	0.49–1.29
rADCmin	Invasive pituitary adenomas	22	0.74 ± 0.12	0.73 (0.67; 0.81)	0.54–1.08
rADCmin	Meningiomas	16	0.95 ± 0.21	0.90 (0.82; 1.03)	0.62–1.41
rADCmin	Prolactinomas	13	0.77 ± 0.23	0.69 (0.59; 0.85)	0.51–1.23
rADCmin	Adamantinomatous craniopharyngiomas	12	1.83 ± 0.44	1.92 (1.56; 2.23)	1.13–2.38
rCBVmax	Non-functional solid adenomas	25	3.37 ± 1.99	3.27 (1.85; 4.36)	0.42–7.50
rCBVmax	Invasive pituitary adenomas	22	4.03 ± 2.23	3.98 (1.96; 5.47)	1.29–8.84
rCBVmax	Meningiomas	16	7.05 ± 3.34	5.82 (5.17; 7.41)	3.78–17.77
rCBVmax	Prolactinomas	13	4.04 ± 1.53	3.75 (2.81; 5.04)	2.01–7.08
rCBVmax	Adamantinomatous craniopharyngiomas	12	2.22 ± 0.95	2.15 (1.84; 2.54)	0.58–3.97
rCBV	Non-functional solid adenomas	25	2.62 ± 1.52	2.55 (1.28; 3.65)	0.29–5.97
rCBV	Invasive pituitary adenomas	22	3.03 ± 1.76	2.60 (1.33; 4.57)	0.97–5.88
rCBV	Meningiomas	16	5.23 ± 2.16	4.53 (3.90; 6.32)	2.53–11.33
rCBV	Prolactinomas	13	2.84 ± 1.21	2.39 (1.78; 3.83)	1.46–4.91
rCBV	Adamantinomatous craniopharyngiomas	12	1.52 ± 0.77	1.39 (1.22; 1.84)	0.39–2.96
rPHmax	Non-functional solid adenomas	25	2.09 ± 1.83	1.40 (0.85; 2.57)	0.29–6.96
rPHmax	Invasive pituitary adenomas	22	2.57 ± 1.27	2.12 (1.70; 3.54)	0.86–5.18
rPHmax	Meningiomas	16	3.43 ± 1.99	3.00 (2.28; 4.50)	0.62–8.16
rPHmax	Prolactinomas	13	3.01 ± 1.51	2.83 (1.84; 4.08)	0.72–6.59
rPHmax	Adamantinomatous craniopharyngiomas	12	1.80 ± 0.89	1.80 (1.45; 2.05)	0.54–4.02
rPH	Non-functional solid adenomas	25	1.63 ± 1.48	1.21 (0.57; 2.18)	0.20–5.92
rPH	Invasive pituitary adenomas	22	1.84 ± 1.03	1.65 (1.14; 2.45)	0.46–4.16
rPH	Meningiomas	16	2.26 ± 1.28	2.10 (1.49; 2.45)	0.49–5.60
rPH	Prolactinomas	13	2.02 ± 1.16	1.75 (1.40; 2.65)	0.29–4.21
rPH	Adamantinomatous craniopharyngiomas	12	0.89 ± 0.50	0.77 (0.47; 1.24)	0.26–1.86

Abbreviations: ADC—apparent diffusion coefficient; rADC—relative apparent diffusion coefficient; rCBV—relative cerebral blood volume; rPH—relative peak height; min—minimum; max—maximum; M—mean; SD—standard deviation; Me—median; Q1—first quartile; Q3—third quartile.

**Table 3:** Diagnostic performance metrics from ROC analysis for combined DWI and PWI parameters in differentiating selected tumor pairs.

Meningiomas vs. Solid Non-Functional Adenomas						
**ADCmin and rPHmax**	threshold	auc.CI	sensitivity	specificity	accuracy	*p*
ADCmin	0.54	0.675 (0.515; 0.818)	1	0.32	0.59	0.050
rPHmax	1.62	0.740 (0.578; 0.880)	0.88	0.6	0.71	0.031
ADCmin and rPHmax		**0.818 (0.690; 0.920)**	0.88	0.76	0.8	0.000
**Meningioms vs. invasive adenomas**						
**ADCmin and rCBV**	**threshold**	**auc.CI**	**sensitivity**	**specificity**	**accuracy**	***p***
ADCmin	0.64	0.872 (0.743; 0.972)	0.81	0.82	0.82	0.000
rCBV	2.83	0.790 (0.625; 0.918)	0.94	0.59	0.74	0.001
ADCmin and rCBV		0.830 (0.699; 0.946)	0.75	**0.91**	0.84	0.000
						
**ADCmin and rCBVmax**	**threshold**	**auc.CI**	**sensitivity**	**specificity**	**accuracy**	***p***
ADCmin	0.64	0.872 (0.743; 0.972)	0.81	0.82	0.82	0.000
rCBVmax	4.54	0.804 (0.659; 0.923)	0.94	0.59	0.74	0.001
ADCmin and rCBVmax		0.815 (0.676; 0.923)	0.81	0.82	0.82	0.000
						
**rADCmin and rCBV**	**threshold**	**auc.CI**	**sensitivity**	**specificity**	**accuracy**	***p***
rADCmin	0.83	0.824 (0.670; 0.952)	0.75	0.82	0.79	0.000
rCBV	2.83	0.790 (0.625; 0.918)	0.94	0.59	0.74	0.001
rADCmin and rCBV		0.767 (0.633; 0.892)	0.62	**0.91**	0.79	0.000
						
**rADCmin and rCBVmax**	**threshold**	**auc.CI**	**sensitivity**	**specificity**	**accuracy**	***p***
rADCmin	0.83	0.824 (0.670; 0.952)	0.75	0.82	0.79	0.000
rCBVmax	4.54	0.804 (0.659; 0.923)	0.94	0.59	0.74	0.001
rADCmin and rCBVmax		0.753 (0.608; 0.892)	0.69	0.82	0.76	0.001
**Meningiomas vs. adamantinomatous craniopharyngiomas**						
**rADCmin and rCBV**	**threshold**	**auc.CI**	**sensitivity**	**specificity**	**accuracy**	***p***
rADCmin	1.13	0.964 (0.891; 1.000)	0.81	1	0.89	0.000
rCBV	3.26	0.984 (0.938; 1.000)	0.88	1	0.93	0.000
rADCmin and rCBV		0.906 (0.812; 1.000)	0.81	1	0.89	0.000
						
**rADCmin and rPH**	**threshold**	**auc.CI**	**sensitivity**	**specificity**	**accuracy**	***p***
rADCmin	1.13	0.964 (0.891; 1.000)	0.81	1	0.89	0.000
rPH	1.53	0.891 (0.750; 0.990)	0.75	0.92	0.82	0.000
rADCmin and rPH		0.844 (0.719; 0.938)	0.69	1	0.82	0.000
						
**rADCmin and rCBVmax**	**threshold**	**auc.CI**	**sensitivity**	**specificity**	**accuracy**	***p***
rADCmin	1.13	0.964 (0.891; 1.000)	0.81	1	0.89	0.000
rCBVmax	4.33	0.995 (0.969; 1.000)	0.94	1	0.96	0.000
rADCmin and rCBVmax		0.906 (0.812; 1.000)	0.81	1	0.89	0.000

**Abbreviations:** ADC—apparent diffusion coefficient; rADC—relative apparent diffusion coefficient; rCBV—relative cerebral blood volume; rPH—relative peak height; min—minimum; max—maximum; M—mean; SD—standard deviation.

## Data Availability

The MRI data used in this study contain sensitive patient information and cannot be shared publicly due to ethical and privacy restrictions. De-identified data used for analysis are available from the corresponding author upon reasonable request and pending institutional approval.
